# Using ACGME milestones as a formative assessment for the internal medicine clerkship: a consecutive two-year outcome and follow-up after graduation

**DOI:** 10.1186/s12909-024-05108-8

**Published:** 2024-03-05

**Authors:** Hsiao-Ju Lin, Jhong-Han Wu, Wei-Hung Lin, Kai-Wen Nien, Huei-Ting Wang, Pei-Jen Tsai, Chiung-Yu Chen

**Affiliations:** 1 Department of Internal Medicine, National Cheng Kung University Hospital, College of Medicine, National Cheng Kung University, Tainan, Taiwan; 2 Education Center, National Cheng Kung University Hospital, College of Medicine, National Cheng Kung University, Tainan, Taiwan

**Keywords:** ACGME Milestones, Clerkship, Internal Medicine

## Abstract

**Background:**

This study evaluated the utility of using Accreditation Council for Graduate Medical Education (ACGME) Milestones as a formative assessment tool for the fifth- and sixth-grade medical students’ performance in their internal medicine (IM) clerkship and the same students’ performance in their post-graduate year (PGY) IM training.

**Methods:**

Retrospective data were collected from 65 medical students completing the two-year IM clerkship in the academic years 2019 and 2020 and 26 of the above students completing their PGY-1 training at the same university hospital in the academic year 2021. Data included the assessment results of 7 of the ACGME IM Milestones, information on admitted patients assigned to the students, and surveys of the students’ satisfaction.

**Results:**

The analysis included 390 assessment results during the IM clerkship and 78 assessment results during the PGY-1 training. Clinical teachers commonly rated level 3 to medical students in the IM clerkship, with PC-2 subcompetency receiving the lowest rating among seven subcompetencies. The levels of most subcompetencies showed stationary in the two-year IM clerkship. Significant improvement was observed in all subcompetencies during the PGY-1 training. The medical students in the second-year IM clerkship expressed higher satisfaction with implementing Milestones than in their first-year IM clerkship and perceived Milestones assessments’ usefulness as learning feedback.

**Conclusions:**

Using ACGME Milestones as a formative assessment tool in the IM clerkship yielded promising outcomes. Longitudinal follow-up of subcompetencies facilitated tracking students’ development and providing constructive feedback.

## Background

With the reform of medical education, competency-based medical education (CBME) has evolved into an outcome-based and learner-centered approach [1, 2]. In this approach, learners engage in experiential learning, receive constructive feedback, and participate in reflective practices to continually refine their skills and knowledge, ultimately acquiring the necessary competencies. Assessments, therefore, play a crucial role in CBME to ensure the sequenced progression and final achievement of competencies by learners. In 2013, the Accreditation Council for Graduate Medical Education (ACGME) and the American Board of Internal Medicine introduced the Internal Medicine (IM) Milestones, breaking the six core competencies into 22 subcompetencies [3]. Each subcompetency is categorized into five levels, describing expected behavioral progress ranging from critical deficiency to the ability to work independently and ultimately achieve aspirational levels. By defining the expected behavioral progress at each level, the Milestones provide explicit references for assessing learners’ competencies, outline the behaviors required for advancement in medical training, and serve as both summative and formative assessments.

Emerging evidence from the United States indicates that milestone-based assessments are a viable approach for evaluating the competency of medical students, as they provide a succinct summary of their performance [4–8]. The Vanderbilt University School of Medicine has developed and applied milestones for a set of focused competencies within the curriculum [4]. It indicated that milestone-based assessment has significant potential to guide medical students’ development. The Johns Hopkins School of Medicine has developed a milestone template to capture the unique characteristics of the genetics elective, and this template received positive feedback from students participating in the curriculum [5]. The University of Michigan Medical School has successfully designed 24 milestones specifically tailored to assess fourth-year medical students during their emergency medicine (EM) clerkship [6]. This outcome highlighted the value and significance of developing a valid and reliable method for evaluating the performance of medical students. Likewise, the University of South Florida Morsani College of Medicine found that utilizing ACGME EM Milestones can effectively identify medical students requiring remediation [7]. The University of Michigan Medical School also reported that 12 of the 16 subcompetencies of ACGME General Surgery Milestones could be utilized to assess the longitudinal development of competencies from surgery clerkship to surgery internship [8]. These individual reports provide evidence supporting the suitability of using milestone-based assessment in undergraduate medical education (UME). Based on these findings, selecting specific subcompetencies of the ACGME IM Milestones may be feasible to assess medical students’ daily observed clinical activities during their IM clerkship. This way, clinical teachers can provide targeted feedback while assessing medical students’ performance in these areas.

Learners exhibited notably different patterns of progress, depending on the specific subcompetency under assessment [9]. Since 2019, 22 subcompetencies of the ACGME IM Milestones have been successfully implemented in assessing post-graduate year (PGY)-2 residents in their IM training and IM residents in 6 teaching hospitals in Taiwan [10]. Through the detailed application of the ACGME IM Milestones to the clerkship, the assessment outcomes can reflect learners’ progress toward competence, highlighting diverse learning paths. Recognizing the points at which learners’ developmental trajectories diverge can serve as potential opportunities for remediation within the context of these subcompetencies [9]. Furthermore, applying the ACGME IM Milestones in clerkship can promote the alignment of educational objectives and assessment methods across different stages of medical training and facilitate the seamless extension of CBME from UME to graduate medical education (GME).

The objectives of this article are fourfold: (1) to choose readily observable competencies from the ACGME IM Milestones, specifically emphasizing patient care (PC) and medical knowledge (MK), (2) to evaluate medical students and analyze the assessment results; (3) to relay the feedback received from medical students who were observed during their two-year clerkship; and (4) to trace the developmental progress of students who continued their training with an additional first-year PGY program at the same university hospital. This study assesses the feasibility of utilizing Milestones as a formative assessment tool to bridge the transition between UME and GME.

## Methods

### Students, post-graduate year-one residents, and clinical teachers

From September 2019 to June 2021, 65 medical students from a medical college in southern Taiwan participated in an IM clerkship program. All the students were in a class cohort, and no one opted out during the two-year clerkship. The two-year IM clerkship program consisted of a twelve-week course divided into two six-week rotations within each year of the clerkship. During the six-week rotation each year, they rotated through diverse subspecialties every two weeks. In the first year, the specialty rotations included sections on gastroenterology, cardiovascular disease, and pulmonology. In the second year, in addition to rotating in the nephrology section, students could choose two other elective subspecialties, such as general medicine and infectious disease, for their learning course.

Of these 65 medical students, 26 underwent PGY-1 training in the same university hospital after graduation. They rotated in the Department of IM for three months as part of their training. It allowed us to closely observe and trace their development regarding their subcompetencies in the ACGME IM Milestones.

The IM department comprised 107 attending physicians, of which 60 had completed the general medicine teacher training conducted by the Taiwan Association of Medical Education. Before implementing Milestones assessments within the Department of IM, we organized two training sessions to elucidate the objectives and procedures of Milestones assessment. In line with the original design by ACGME, our institution adopted Milestones for assessing the competency of PGY residents in August 2018. When we extended Milestones assessments to IM clerkship, all the attending physicians already had a year of experience with Milestones assessments.

### Clinical activities of clerkship

During the IM clerkship, medical students assumed the role of frontline providers of patient care. Each medical student was mentored by an attending physician and received close supervision and guidance from experienced residents and the attending physician while rotating through each subspecialty. One of their primary responsibilities was documenting all medical records, including admission notes or on-service notes, progress notes, weekly summaries, and discharge or off-service notes if patients could not be discharged at the end of the subspecialty rotation. In addition, medical students practiced bedside skills for their assigned patients. The level of supervision varied depending on the complexity and significance of the task. While students were encouraged to propose diagnostic and therapeutic plans, the supervising physicians must agree to and sign those medical orders before execution. The supervising physicians ultimately retained the overall responsibility for ensuring the quality of care.

During the IM rotations, medical students participated in various assessment activities. The supervising attending physicians conducted short practice observation assessments every two weeks, which included 1 to 2 mini-clinical evaluation exercise (mini-CEX) assessments and audits of the students’ patient care documentation. Furthermore, medical students presented their assigned cases to the care team during ward rounds, demonstrating their understanding of the patient’s condition and ability to communicate effectively within the team.

### The Taiwanese version of IM milestones

The original ACGME IM Milestones 1.0 version, introduced in 2013–2014, presented expected behavioral descriptions for each subcompetency along the developmental continuum [3]. However, some descriptions employed complex language, predominantly laden with educational jargon [11]. This complexity made it challenging for users to comprehend the descriptions, increasing the time and difficulty involved in the assessment. For the convenience of Taiwanese users, we used the Taiwanese 1.0 version of the IM Milestones in this study, which was translated into Chinese through a collaborative effort between the Taiwan Society of Internal Medicine and educational experts [10]. This translation employed language that was readily comprehensible to both students and teachers to delineate the expected behaviors for each level of competency. Furthermore, our electronic portfolio (e-portfolio) system included additional explanations for specific behavioral descriptions through pop-out windows, enhancing clarity and making the assessment process and feedback more accessible.

### Using milestones to assess learning outcomes and provide feedback

Based on the aforementioned clinical activities, we have selected seven of the 22 subcompetencies outlined in the ACGME IM Milestones to assess students’ performance. These subcompetencies were PC-1 (gathering information for defining the problem), PC-2 (management planning), PC-4 (bedside skill), MK-1 (clinical knowledge), MK-2 (diagnostic knowledge), systems-based practice (SBP)-4 (patient transition), and professionalism (PROF)-1 (professional and respectful interaction).

Each attending physician evaluated their supervised student’s progress and performed Milestones assessments according to the student’s performance in the clinical activities at the end of each specialty rotation. They utilized the Taiwanese 1.0 version of the IM Milestones worksheet on the e-portfolio system and selected statements accurately matching the students’ behaviors during the rotation. The e-portfolio system automatically determined the level of competence based on the selected statements. Following the original ACGME design, the competence levels were scored on a scale of 1 to 5, with increments of 0.5. Each level represented a different degree of competence, ranging from critical deficiency to aspirational performance [3]. The behavior statements chosen by the attending physician and the system’s assigned levels were displayed on the e-portfolio system. It allowed students to compare these two on the e-portfolio system directly every two weeks at the end of each subspecialty rotation and the sixth week after completing the IM rotation. It provided insights into areas that require improvement. Furthermore, students could engage in discussions with their supervising attending physicians on strategies for attaining advanced-level behaviors.

In total, medical students underwent Milestones assessments six times, with one assessment conducted every two weeks during the six-week rotation in both the first- and second-year IM clerkships. Instead of being used for summative decisions, the Milestones assessments conducted for medical students and PGY-1 residents were used as formative assessments. We defined level 1 of Milestones (i.e., the level of critical deficiency) as a low Milestones rating for medical students. It would necessitate further investigation to understand the underlying causes and determine the appropriate solutions to assist the student.

During the three-month IM rotation of the first-year PGY training, each PGY-1 resident received a Milestones assessment from their supervising attending physician at the end of each month, resulting in three assessments throughout the training. The assessment encompassed all 22 subcompetencies of the Taiwanese 1.0 version of the IM Milestones, including the seven subcompetencies selected for medical students.

### The learning curve

To illustrate the diverse learning trajectories among the 26 students who completed their PGY-1 training at our hospital after graduation, we compiled a learning curve using the final Milestones assessment ratings during their first- and second-year IM clerkship and PGY-1 training.

### The straight line scoring

Straight line scoring (SLS), a string of identical ratings, is when a single learner receives the same score on the 9-point scale across all Milestones subcompetencies [12]. Given that the progress of learning in each subcompetency of Milestones typically varies among learners, achieving an SLS purely by chance is highly unlikely. Assuming that a resident was accurately rated in each subcompetencies of Milestones, an SLS would seldom occur. Following the ACGME method for evaluating the results of the Milestones assessment, we checked the SLS rate of our assessment results. We also calculated the rated level of these SLSs.

### The characteristics of the patients

Transitioning to new contexts poses challenges for medical students as competent performance is context-dependent [13]. The complexity of patients may impact the student’s learning experience and performance. We recorded the number of patients assigned to medical students during each specialty rotation to trace the development of their patient care capability. To capture contextual information, we categorized the cause of admission into two groups: patients admitted for scheduled procedures or treatments and patients admitted for acute illness. We also recorded the length of inpatient hospital stay and whether a handover was required at the end of the subspecialty rotation.

### End-of-rotation surveys

Upon completing the first- and second-year IM clerkships, students must complete a satisfaction survey via the e-portfolio system. This survey employed a Likert scale featuring five response options, namely 1 (strongly disagree), 2 (disagree), 3 (neutral), 4 (agree), and 5 (strongly agree). The survey encompassed a wide range of aspects, including overall satisfaction with the IM clerkship, the extent to which the Milestones assessment results align with their self-evaluation results, and the perceived usefulness of Milestones assessment as a feedback mechanism for their ongoing learning.

### Statistical analysis

Following the approval of the Institutional Review Board of National Cheng Kung University Hospital, considered to be an expedited review (A-ER-111-473-T), we retrospectively collected the data mentioned above of the 65 medical students who underwent IM rotations in the academic years 2019 and 2020 and the data of these 26 PGY-1 residents who underwent the IM training courses in the academic year 2021. The data during 2019/08/01-2022/07/31 that the Institutional Review Board of National Cheng Kung University Hospital waived the participant informed consent requirement included the medical records written by the medical students in their IM rotations, the mini-CEX, and the Milestones assessment results of students and PGY residents in their IM training courses.

The progress in the Milestones level of seven subcompetencies of the same student from the two-year IM clerkship and then the PGY-1 IM training were analyzed using the Friedman test. The difference in ratings among the seven subcompetencies of the Milestones and the seven categories of the mini-CEX were assessed using the Kruskal-Wallis test, followed by Dunn’s post hoc test. The effect sizes between students in the first- or second-year clerkship and PGY-1 training were calculated by Cohen’s d. The difference in categorical variables between the first- and second-year IM clerkship, such as the rate of SLS, the reason for hospitalization, and the rate of patients needing a handover, were compared using the Chi-square test. The difference in the patient number and the length of hospital stay of patients assigned to medical students between the first- and second-year IM clerkship were assessed using the paired t-test and the independent t-test, respectively. The Wilcoxon signed-rank test analyzed the changes in scores in the end-of-rotation survey between the first- and second-year IM clerkship. Two-tailed analytical results with *p*-values less than 0.05 were regarded as statistically significant. Data were analyzed using SPSS Statistics for Windows, Version 23.0 (IBM SPSS Statistics for Windows, Version 23.0. Armonk, NY: IBM Corp).

## Results

### Number of milestones and mini-CEX assessments and chart audit – the database

During the two-year IM clerkship, 65 medical students completed one Milestones assessment in each subspecialty rotation, resulting in 390 assessment results available for analysis. During this period, they underwent 273 mini-CEX assessments and 424 chart audits in the first-year IM clerkship. In the second-year clerkship, they completed 207 mini-CEX assessments and 417 chart audits. Furthermore, 26 PGY-1 residents underwent monthly assessments for three months and yielded 78 assessment results to trace their progress during the early stage of their medical careers.

### Straight line scoring of milestones assessment results – the quality of assessment

In the first-year IM clerkship, the SLS rate was 36.9%. Among these SLSs, the majority (86.1%) were rated at level 3, followed by levels 4 and 2, accounting for 8.3% and 5.6%, respectively. The SLS rate in the second-year IM clerkship was 17.4%, significantly lower than in the first-year IM clerkship (*p* < 0.0001). Like in the first-year IM clerkship, level 3 was the most commonly rated, accounting for 88.2%. The remaining SLSs were allocated to levels 2, 3.5, and 4, representing 2.9%, 2.9%, and 5.9%. The SLS rate in the PGY-1 training was 6.4%, significantly lower than in the first-year clerkship (*p* < 0.0001), and all the SLSs were rated at level 4.

### The distribution of rated levels in each subcompetency of milestones – the global view of assessment outcomes

Attending physicians most frequently rated level 3 across the clerkship’s seven subcompetencies of the Milestones. In the first-year IM clerkship, approximately 66.2% of assessment results were rated as level 3, with only 17.0% being rated below level 3. Similarly, around 66.0% of the second-year IM clerkship assessment results for the seven subcompetencies were rated level 3, while 17.4% were rated below 3.

One first-year clerkship student received six level-1 ratings in seven Milestones subcompetencies, except for the PC-4 subcompetency. However, upon follow-up, this student improved and progressed in all seven subcompetencies during the second-year IM clerkship. In the second-year IM clerkship, only a level-1 rating was observed for the PC-2 subcompetency of another medical student.

### The scores for each category of mini-CEX Assessment – another evaluation metric

The mean scores of mini-CEX for each year of clerkship were presented in Table 1. In the first-year clerkship, the mean score for the seven categories ranged from 4.3 to 4.5, with no significant difference. In the second-year clerkship, the mean score went from 4.4 to 4.7. A significant difference in scoring was observed between the categories of medical interviewing and informed decision-making/counseling, with scores of 4.7 ± 0.7 and 4.4 ± 0.6, respectively (*p* = 0.016).

**Table 1:** The Comparison of Scoring of Mini-CEX in the Same Year Clerkship

*N* = 65	First-year clerkship	Second-year clerkship
Medical interviewing	4.4 ± 0.6	4.7 ± 0.7^a^
Physical examination	4.3 ± 0.7	4.4 ± 0.7
Informed decision-making/Counselling	4.4 ± 0.6	4.4 ± 0.6^a^
Clinical judgement/Reasoning	4.4 ± 0.6	4.5 ± 0.6
Organization/Efficiency	4.4 ± 0.6	4.4 ± 0.6
Professionalism/Humanity	4.5 ± 0.6	4.5 ± 0.6
Overall clinical competency	4.4 ± 0.6	4.6 ± 0.6

Data was expressed as mean ± standard deviation. The same alphabet indicates significant differences between the indicated competencies in the same year of learning by the Kruskal-Wallis test with Dunn’s post hoc test

### The comparison of ratings among seven subcompetencies – the development of milestones subcompetencies

As shown in Table 2, the PC-2 subcompetency received the lowest rating with a mean level of 2.81 ± 0.64 among the seven subcompetencies in the first-year IM clerkship. Except for the MK-2 subcompetency, there were significant differences between the PC-2 subcompetency and the other five subcompetencies (*p* < 0.000001). In the second-year IM clerkship, PC-2 remained the subcompetency with the lowest rating, with a mean level of 2.65 ± 0.65. The SBP-4 subcompetency had the highest rating among the seven subcompetencies, with a mean of 3.20 ± 0.40. The PC-2 subcompetency and the SBP-4 subcompetency significantly differed from the other six subcompetencies (*p* < 0.000001).

**Table 2:** The comparison of ratings among seven subcompetencies in the same-year clerkship

*N* = 65	First-year clerkship	Second-year clerkship
PC-1	3.00 ± 0.55^a^	3.05 ± 0.47^g,l^
PC-2	2.81 ± 0.64^a,b,c,d,e,f^	2.65 ± 0.65^g,h,i,j,k^
PC-4	3.04 ± 0.48^b^	2.98 ± 0.45^h,m^
MK-1	3.02 ± 0.49^c^	3.01 ± 0.40^i,n^
MK-2	2.96 ± 0.50^d^	2.99 ± 0.44 ^o^
SBP-4	3.09 ± 0.45^e^	3.20 ± 0.40^j,l,m,n,o,p^
PROF-1	3.07 ± 0.51^f^	3.05 ± 0.42^k,p^

Data was expressed as mean ± standard deviation. The same alphabet indicates significant differences between the indicated competencies in the same year of learning by the Kruskal-Wallis test with Dunn’s post hoc test. Abbreviations: PC patient care, MK medical knowledge, SBP systems-based practice, PROF professionalism

### The progress of subcompetencies within the two-year clerkship and to PGY-1 training – the learning trajectory

When comparing the levels of seven subcompetencies between the first- and second-year IM clerkships, we found that the rated levels did not show significant changes except for the PC-2 subcompetency and the SBP-4 subcompetency (Table 2). The PC-2 subcompetency showed a significantly lower rating in the second-year IM clerkship than in the first-year IM clerkship (*p* = 0.015); the SBP-4 subcompetency showed a significantly higher rating in the second-year IM clerkship than in the first-year IM clerkship (*p* = 0.017).

Among the 26 PGY-1 residents, the Milestones assessment results during their IM clerkship were similar to those of other students, with the PC-2 subcompetency still being the lowest mean level (2.91 ± 0.64 and 2.74 ± 0.69, respectively). During the PGY-1 training, a significant improvement in all seven subcompetencies of these 26 PGY-1 residents was noted. The Cohen’s d values calculated between the first-year clerkship students and PGY-1 residents and between the second-year clerkship students and PGY-1 residents indicated large effect sizes (Table 3). Attending physicians rated PGY-1 residents higher than the medical students across all seven subcompetencies. The majority of the ratings for PGY-1 residents fell within the range of levels 3.0 (45.4%), 3.5 (11.5%), and 4.0 (36.2%). However, the PC-2 subcompetency still had a lower mean level (3.29 ± 0.67) than other subcompetencies (Table 3).

**Table 3:** The progress of seven subcompetency from the two-year clerkship to PGY-1 training

*N* = 26	1st	2nd	PGY-1	*p*-value*	Cohen’s d	
					1st -PGY1	2nd -PGY
PC-1	3.06 ± 0.52^a^	3.10 ± 0.46^b^	3.51 ± 0.61^a,b^	< 0.0001	0.79	0.76
PC-2	2.91 ± 0.64^c^	2.74 ± 0.69^d^	3.29 ± 0.67^c,d^	< 0.0001	0.58	0.81
PC-4	3.10 ± 0.44^e^	3.01 ± 0.40^f^	3.42 ± 0.52^e,f^	< 0.0001	0.65	0.89
MK-1	3.06 ± 0.49^g^	3.08 ± 0.33^h^	3.37 ± 0.56^g,h^	0.001	0.59	0.66
MK-2	2.99 ± 0.54^i^	2.96 ± 0.42^j^	3.33 ± 0.49^i,j^	< 0.0001	0.66	0.82
SBP-4	3.12 ± 0.40^k^	3.20 ± 0.36^l^	3.67 ± 0.49^k,l^	< 0.0001	1.21	1.09
PROF-1	3.09 ± 0.45^m^	3.08 ± 0.40^n^	3.57 ± 0.54^m,n^	< 0.0001	0.96	1.05

Data was expressed as mean ± standard deviation. The same alphabet indicates significant differences between the same subcompetency of the different learning periods; *By Friedman test. Abbreviations: 1st Frist-year clerkship; 2nd Second-year clerkship; PGY-1 Post-graduate year 1; PC patient care, MK medical knowledge, SBP systems-based practice, PROF professionalism

**Fig. 1:**
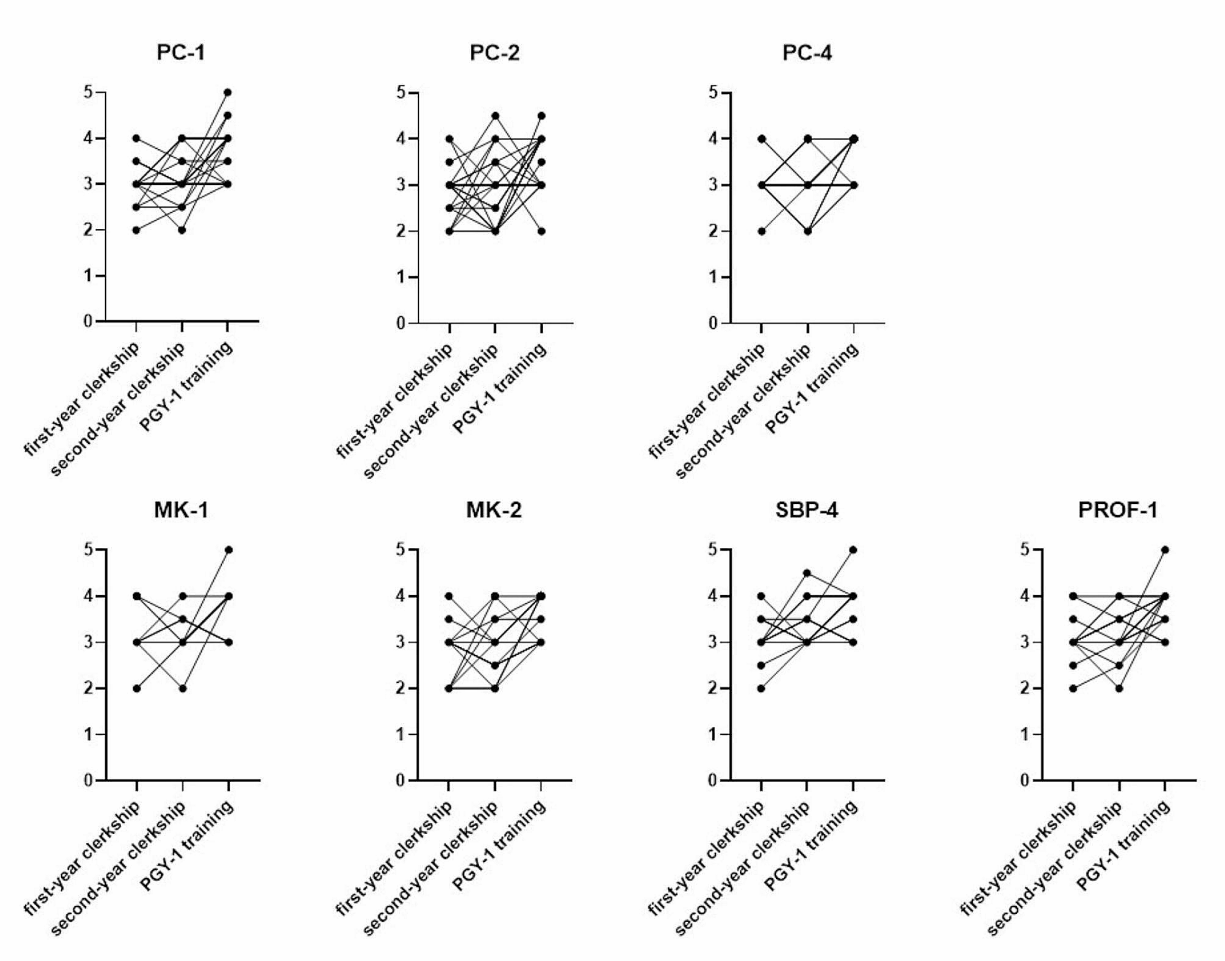
The overlaid learning curves for the seven subcompetencies of the 26 medical students who completed their PGY-1 training at our hospital. Abbreviations: PC-1 patient care-1, PC-2 patient care-2, PC-4 patient care-4, MK-1 medical knowledge-1, MK-2 Medical knowledge-2, SBP-4 systems-based practice-4, PROF-1 professionalism-1

In Fig. 1, the overlaid learning curves depicted a consistent trend of increasing competency from the clerkship to PGY-1 training. It was worth noting that for all seven subcompetencies, the variation in competency levels between individuals either remained constant or narrowed. There was an exception in the PC-2 subcompetency curve; the PC-2 competency levels became diverse, and one PGY-1 resident exhibited regression.

### The characteristics of patients assigned to medical students – the context of learning

Approximately six patients were assigned to medical students in the first-year IM clerkship, and there was no significant progress in patient numbers cared for by the second-year IM clerkship students (6.4 ± 2.2 vs. 6.3 ± 1.8, *p* > 0.05). Most patients assigned to students in the first-year IM clerkship were hospitalized for scheduled events (56.5%). In contrast, only 7.3% of patients assigned to the second-year IM clerkship students were hospitalized for scheduled events, and 92.7% were admitted due to acute illness. Irrespective of the reason for hospitalization, patients cared for by the second-year IM clerkship students tended to have a more extended inpatient hospital stay than those managed for by the first-year IM clerkship students. The mean inpatient hospital stay was 5.6 days in the first-year IM clerkship and 12.2 days in the second year (*p* < 0.0001). As a result, clinical handovers at the end of specialty rotation were more frequently encountered in the second-year IM clerkship, with 10.2% of patients experiencing a care transfer from one student to another. Only 0.2% of patients cared for by the first-year IM clerkship students had a similar experience.

### The feedback of medical students on the milestones assessment – the perspective of students

All 65 students completed the questionnaires with a 100% response rate for the two-year IM clerkship. The mean satisfaction scores for the IM clerkship in the first and second years were more than 4, and the difference was not statistically significant (*p* > 0.05). The satisfaction with the Milestones assessment increased significantly in the second-year IM clerkship. The mean scores for the usefulness of Milestones as feedback for learning and the consistency between results of Milestones assessment and self-assessment were considerably higher in the second-year clerkship than in the first-year clerkship (4.1 ± 0.9 vs. 3.6 ± 0.8, *p* < 0.0001 and 4.2 ± 0.8 vs. 3.5 ± 0.7, *p* < 0.0001, respectively).

## Discussion

By selecting specific subcompetencies of the ACGME IM Milestones to assess the daily clinical activities of medical students, we successfully applied the Milestones as a formative assessment for the IM clerkship. As anticipated, our study demonstrated that the subcompetency levels of medical students were generally around 3, indicating they were progressing and improving their performance as defined by the ACGME IM Milestones 1.0 version [3]. The assessment results also disclosed the weakness of medical students in their performance. Additionally, our study revealed that the Milestones assessment effectively illustrated the trajectory of competence development from the stage of medical students to PGY-1 residents.

By incorporating the Reporter, Interpreter, Manager, Educator (RIME) model and the Core Entrustable Professional Activities (EPAs) framework, 13 core EPAs for preparing students to enter residency have been introduced in UME in the United States [14]. The analysis of the results of the EPAs assessment identifies three clusters of EPAs [15]. These clusters consist of EPAs that align well with existing curricula and provide limited opportunities for practice due to their infrequent occurrence and some EPAs that need to be included or developed in the current curricula. This result emphasized aligning workplace-based assessment contents with the curriculum for meaningful evaluation. For our IM clerkship, we selected relevant subcompetencies from the ACGME IM Milestones to assess the daily activities of medical students, such as gathering information for defining the problem (PC-1), management planning (PC-2), bedside skills (PC-4), clinical knowledge (MK-1), and diagnostic knowledge (MK-2). Additionally, we aimed to foster team collaboration and professional behaviors by incorporating patient transition (SBP-4) and professional and respectful interaction (PROF-1) as learning objectives, recognizing that assessment drives learning. The contents of the RIME model encompass gathering a history and performing a physical examination, documenting a medical record, providing an oral presentation, prioritizing a differential diagnosis, interpreting common diagnostic and screening tests, and recognizing an urgent or emergency patient as EPAs designated for reporters and interpreters [14]. This arrangement in choosing these daily activities for assessing medical students aligns with our strategy in selecting observable subcompetencies in this study.

As part of the National Accreditation System, Milestones ratings were expected to vary by subcompetency, assuming independent assessment of each subcompetency’s performance. The SLS rate was considered an indicator of assessment quality [12]. Our study found a decreasing SLS rate from the clerkship to PGY-1 training, with medical students predominantly rated at level 3 and PGY-1 residents more frequently rated at level 4. These discrepancies in rated levels may be explained by clinical teachers’ preconceived notions of the overall competence of medical students and PGY-1 residents or the halo effect, meaning observed competence levels extrapolated to less observed levels. Another plausible explanation might be that teachers were unfamiliar with using Milestones assessments. To further explore the reason, we collected and analyzed data on the SLS rate of the same group of attending physicians for PGY-1 residents in the academic years 2019, 2020, and 2021 (data not shown in the results). We found that the SLS rates were 6.7%, 8.9%, and 9.9%, respectively. As a result, it was less plausible to attribute the decline in the SLS rate to teachers gradually becoming more acquainted with Milestones assessments. We considered that along with students transitioning from the clerkship to PGY training, their duration of IM practice increased. Owing to the increased frequency of clinical activities, the clinical teachers were enabled to observe performance closely, resulting in the improved quality of assessments and a reduced SLS rate.

In Taiwan, the interpretation of each rating scale on the mini-CEX differs from the original version in that a rating of 4 signifies performance that meets the standards expected of clerks, a rating of 5 indicates performance that meets the standards for interns, and a rating of 6 represents performance that meets the standards for residents (or PGY-1 residents). Our mini-CEX assessment results revealed that the ratings for medical students mostly ranged from 4 to 5. In contrast, our Milestones assessment results indicated that students’ subcompetency levels were primarily at level 3, signifying that they were progressing and improving their performance. When attending physicians assessed students using the mini-CEX, they evaluated their performance by comparing it with their peers’ performance or relying on their perceptions of how students should perform. Conversely, attending physicians assessed students’ performance by selecting specific behaviors described in each Milestone level. Although based on different criteria, these two assessments led to similar results, indicating the viability of our approach in selecting observable subcompetencies from the ACGME IM Milestones for a formative assessment.

When comparing the assessment results of the seven subcompetencies, it was evident that the PC-2 subcompetency, which involves applying knowledge learned in the classroom to make diagnostic and therapeutic plans, consistently lagged behind other subcompetencies, from medical students to PGY-1 residents. Similarly, previous studies have reported that many junior doctors and medical students feel ill-prepared when developing care plans [16, 17]. This result suggested the importance of dealing with real-life situations and on-the-job learning to acquire implicit knowledge. Our report showed that the SBP-4 subcompetency received significantly higher ratings in the second-year clerkship compared to other subcompetencies, which may be attributed to the increased hospital stay duration of patients and the frequency of handovers required.

As mentioned by Hauer et al., the context is a crucial factor influencing ad hoc entrustment decisions [18]. When designing an EPA assessment, it is essential to specify the “specifications and limitations” of the task to ensure the scope of the context when assessing this EPA. Milestones assessment lacks descriptions regarding the context at the time of evaluation, which should be a factor to consider when interpreting Milestones assessment results. In the two-year IM clerkship, the characteristics of assigned patients cared for by the students changed from shorter hospital stays and stable conditions to more extended stays and acute illnesses. This change in context may explain the stationary or regress in competency levels. Unlike the patients cared for by the first-year clerkship students, who already had preliminary treatment plans, the students in the second-year clerkship encountered patients needing multiple tests and complex treatment options due to acute conditions, resulting in a decline in the PC-2 subcompetency (management planning).

Assignment of responsibility helps the development of competency. The social cognitive theory suggests that learners should be allowed to observe and model responsible behavior. In addition, the constructivism theory indicates that assignment of responsibility is crucial for learners to develop a deep understanding of concepts and skills. When comparing medical students functioning as frontline care providers under close supervision to PGY-1 residents having obtained their medical licenses, the latter is entrusted with making the majority of decisions independently in primary care. Our IM Milestones assessment tracing showed significant improvements in all seven subcompetencies for the same PGY-1 resident compared to their IM clerkship learning period.

The overlaid learning curves illustrate the complete variation in the learning trajectories of a group of learners within a specific learning domain. Instructors can utilize learning curve information to allocate educational resources to individuals needing support or intervention [19]. Creating a learning curve requires a fine-grained collection of data. Despite utilizing Milestones as a formative assessment to evaluate learners within a short rotation period, our overlaid learning curves still revealed a divergent trajectory in the PC-2 subcompetency. Our findings require a more extended observation to validate the use of such assessment results in constructing learning curves. However, our results may provide learners and instructors with self-directed learning and education management opportunities.

The increased transparency of performance expectations in Milestones offers a comprehensive and structured approach to feedback [20]. Through our Chinese 1.0 version of the IM Milestones assessments using the Chinese spoken language, our students could directly compare behavior descriptions selected by attending physicians with higher-level expectations displayed on the e-portfolio system. It helped them understand areas where improvement was needed and facilitated them to discuss with their supervising attending physicians how to achieve the desired behaviors at an advanced level. Our results showed that the number of Milestones level-1 ratings was reduced from the first-year clerkship to the second-year clerkship. Like the satisfaction of medical students from other schools implementing Milestones, our end-rotation survey revealed that as students became more acquainted with the IM Milestones, there was a notable increase in satisfaction with the feedback received from the results of Milestones assessment and alignment of Milestones assessment results with self-assessment results [4, 5, 8].

Due to the desire for students to have more experience in diverse subspecialties in the IM clerkship, our curriculum design allowed them to rotate every two weeks. Assessing student performance in short rotations presents challenges, and a practical approach, as we did, involves selecting subcompetencies closely linked to the clinical activities that supervising attending physicians can observe daily. Accordingly, we have chosen subcompetencies primarily derived from the competencies of patient care and medical knowledge for our assessment contents. We may excessively focus on the two competencies and need to assess more competencies for implementing CBME. Instead of using Milestones as a summative assessment, we conducted Milestones assessments every two weeks and employed them as a formative assessment, similar to the ad hoc EPAs. Its value as an information source for the clinical competency committee must be further validated. However, considering the 13 core EPAs in UME, not covered by all UME curricula [15], our approach of using the seven instead of all 22 subcompetencies of the ACGME IM Milestones to assess the competencies of medical students is worth continuing. Our finding was a single-hospital experience. Medical education systems vary significantly from country to country, and factors like rotation duration, the ratio of preceptor to student, and the number of patients cared for by the students may also differ. Therefore, when applying our assessment strategy, adapting and validating it according to the unique conditions and specific requirements within each context is essential.

## Conclusions

Our study demonstrated that selecting specific subcompetencies from the ACGME IM Milestones as a formative assessment for medical students is feasible. In addition to giving feedback, these Milestones can also disclose the competence levels of medical students and their developmental trajectories. Implementing the ACGME IM Milestones in clerkship will improve the UME curriculum and align the blueprint for competency development from UME to GME.

## Data Availability

The datasets used and/or analyzed during the current study are available from the corresponding author upon reasonable request.

## Abbreviations

ACGME: Accreditation Council for Graduate Medical Education
CBME: competency-based medical education
EM: emergency medicine
EPAs: entrustable professional activities
e-portfolio: electronic portfolio
GME: graduate medical education
IM: internal medicine
mini-CEX: mini-clinical evaluation exercise
MK: medical knowledge
PC: patient care
PGY: post-graduate year
PROF: Professionalism
SBP: systems-based practice
SLS: straight line scoring
UME: undergraduate medical education
